# Novel method for diagnosing takotsubo syndrome with left ventriculography using a microaxial flow pump in temporary surgical mode in a patient with cardiogenic shock: a case report

**DOI:** 10.1093/ehjcr/ytae578

**Published:** 2024-10-24

**Authors:** Shoji Kawakami, Eigo Nawata, Jun-ichiro Nishi

**Affiliations:** Department of Cardiology, Aso Iizuka Hospital, 3-83 Yoshio-machi, Iizuka, Fukuoka 820-8505, Japan; Department of Cardiology, Aso Iizuka Hospital, 3-83 Yoshio-machi, Iizuka, Fukuoka 820-8505, Japan; Department of Cardiology, Aso Iizuka Hospital, 3-83 Yoshio-machi, Iizuka, Fukuoka 820-8505, Japan

**Keywords:** Takotsubo cardiomyopathy, Cardiogenic shock, Microaxial flow pump, Impella CP, Case report

## Abstract

**Background:**

It is reasonable to introduce a microaxial flow pump (Impella) before coronary angiography and left ventriculography (LVG) to prioritize treating tissue hypoperfusion in patients with takotsubo syndrome (TTS) and refractory cardiogenic shock. However, left ventricular (LV) unloading by an Impella device might prevent contrast media from filling the left ventricle, making it difficult to evaluate LV wall motion abnormalities during LVG.

**Case summary:**

A 76-year-old female with ST elevations in I, II, aVL, and V1–6 on electrocardiography and severe LV dysfunction on echocardiography immediately received circulatory support with Impella CP to treat refractory cardiogenic shock. Subsequent coronary angiography showed no significant stenosis. Biplane LVG was performed using an additional pigtail catheter inserted into the left ventricle while the pump catheter remained there in temporary surgical mode, which was able to protect the motor because the purge system remained active while the pump was stopped. Left ventriculography in temporary surgical mode revealed apical ballooning with a mismatch between epicardial coronary artery perfusion and LV contraction without compromised haemodynamics. The patient was diagnosed with TTS.

**Discussion:**

Coronary angiography and LVG are considered essential diagnostic tools to confirm TTS and exclude acute myocardial infarction. Left ventriculography with Impella temporarily set to surgical mode was able to clearly evaluate LV wall motion abnormalities without affecting haemodynamics. This case highlights that it is perfectly acceptable to prioritize Impella insertion over coronary angiography and LVG in patients with refractory cardiogenic shock in whom the differentiation between TTS and acute myocardial infarction has not yet been made.

**Learning points**

- Urgent coronary angiography and biplane left ventriculography are the gold standard for confirming takotsubo syndrome and excluding ST elevation acute myocardial infarction.
- Immediate introduction of a microaxial flow pump should be considered for patients with worsening cardiogenic shock, before coronary angiography and left ventriculography.
- In patients treated with a microaxial flow pump, left ventriculography in surgical mode allows for accurate assessment of left wall motion abnormalities.

## Introduction

There is currently no widely established non-invasive tool for rapid and reliable diagnosis of takotsubo syndrome (TTS). However, urgent coronary angiography and biplane left ventriculography (LVG) are essential to differentiate TTS from acute myocardial infarction with certainty in patients presenting with ST elevation.^1,2^ However, in patients with refractory cardiogenic shock, induction of mechanical circulatory support might be necessary to improve tissue perfusion without delay. Several studies have reported that early use of a microaxial flow pump (Impella CP; Abiomed, Danvers, MA, USA), which pumps blood from the left ventricle through a catheter and expels it into the ascending aorta, leads to improved early haemodynamics and prognosis for patients with cardiogenic shock due to acute myocardial infarction^3,4^ and TTS.^2,5–8^ However, there are no reports mentioning the quality of LVG for evaluating left ventricular (LV) wall motion abnormalities after the introduction of an Impella device in patients with TTS because LVG has been performed prior to Impella insertion. Herein, we report a case of TTS complicated by refractory cardiogenic shock in which LVG with the Impella device temporarily set to surgical mode was able to clearly evaluate LV wall motion abnormalities without affecting haemodynamics.

## Summary figure

**Figure:**
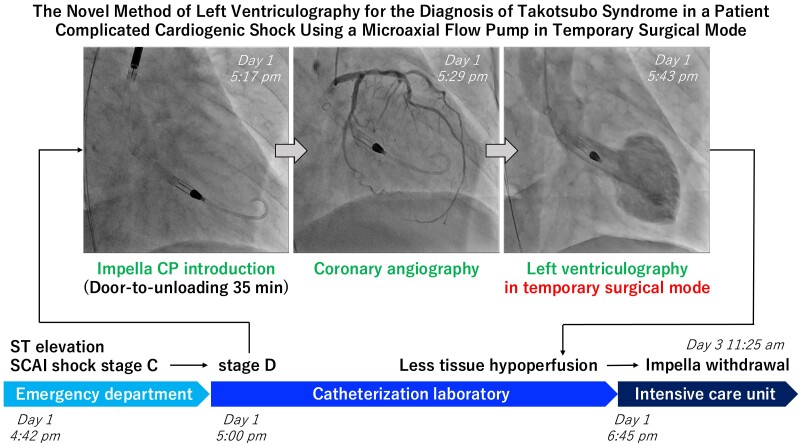


## Case presentation

**Figure 1:**
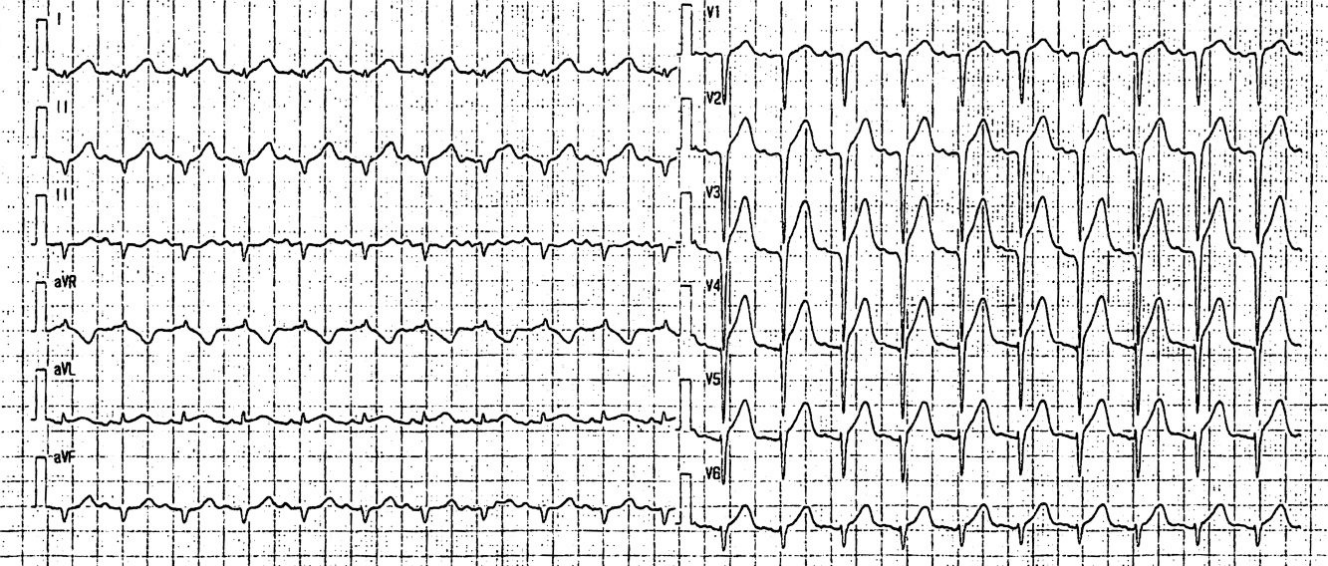
Twelve-lead electrocardiography at hospital admission revealed ST elevations in I, II, aVL, and V1–6.

A 76-year-old woman with a history of hypertension, dyslipidaemia, and diabetes mellitus was transferred to the emergency department with chest pain. On arrival (Day 1, 4:42 p.m.), she was in a clinical state of shock with fatigue, cool extremities, blood pressure of 86/50 mmHg, pulse of 132 b.p.m., respiratory rate of 30/min, oxygen saturation of 99%, and lactate level of 2.1 mmol/L (reference: <1.8 mmol/L). On arrival in the emergency department, her white blood cell count was 21.9 × 10^9^/L (reference: 3.3 × 10^9^–8.6 × 10^9^/L), haemoglobin was 11.1 g/dL (reference: 11.6–14.8/L), platelet count was 295 × 10^9^/L (reference: 158 × 10^9^–348 × 10^9^/L), C-reactive protein was 15.09 mg/dL (reference: <0.14 mg/dL), serum creatinine was 0.61 mg/dL (reference: 0.46–0.79 mg/dL), creatine kinase was 77 IU/L (reference: 41–153/L), cardiac troponin I was 3897.0 pg/mL (reference: <26.2 pg/mL), and brain natriuretic peptide was 62.1 pg/mL (reference: <18.4 pg/mL). Her electrocardiogram revealed ST elevations in I, II, aVL, and V1–6 (*Figure 1*). Echocardiography revealed akinesis of the apex and anterior LV wall with hyperkinesis of the base, LV ejection fraction of 40%, no LV outflow obstruction, no severe valvular disease, no LV thrombi, and no right ventricular dysfunction. She was classified as having Society for Cardiovascular Angiography and Interventions (SCAI) shock stage C. Norepinephrine 0.06 μg/kg/min was started.

**Figure 2:**
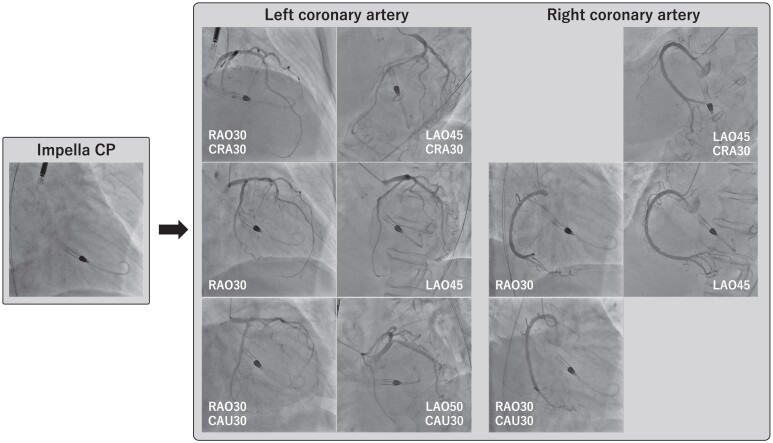
Coronary angiography revealed no significant stenosis of the epicardial coronary artery. LAO, left anterior oblique; RAO, right anterior oblique.

**Figure 3:**
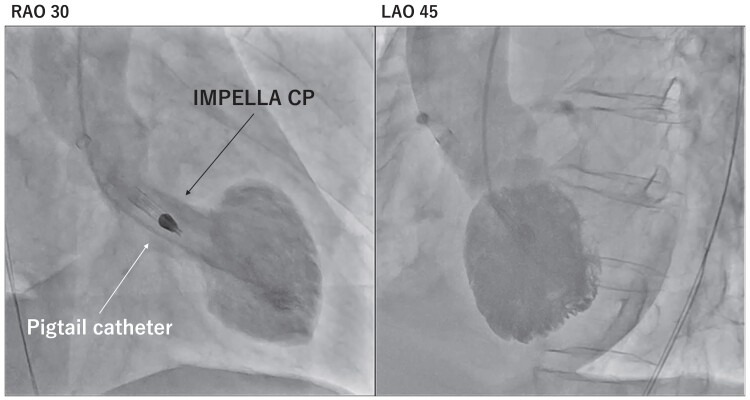
Biplane left ventriculography using an additional pigtail catheter inserted into the left ventricle while the microaxial flow pump catheter remained in the left ventricle while in temporary surgical mode. Left ventriculography revealed apical ballooning with a perfusion-contraction mismatch, suggesting takotsubo syndrome. LAO, left anterior oblique; RAO, right anterior oblique.

In the catheterization laboratory, at 5:00 p.m. on Day 1, her systolic blood pressure dropped to 80 mmHg even with norepinephrine at 0.11 μg/kg/min, indicating SCAI shock stage D. After LV end-diastolic pressure was measured at 25 mmHg and the LV apex was confirmed with a small amount of contrast media injected through a pigtail catheter, Impella CP was introduced at 5:17 p.m. After Impella support began, blood pressure increased to 100 mmHg. At 5:29 p.m., coronary angiography revealed no significant stenosis of the epicardial coronary artery despite persistent chest pain and ST elevations (*Figure 2*). Although LVG had to be performed to confirm the diagnosis of TTS, the contrast media might not have adequately filled the LV because the Impella device draws blood from the left ventricle. Therefore, LVG was performed using an additional pigtail catheter inserted into the left ventricle while the Impella device remained there in temporary surgical mode at 5:43 p.m. (*Figure 3*; Supplementary material online, *Videos S1* and *S2*). Biplane LVG, which was performed with 30 mL of contrast media administered at a rate of 10 mL per second and surgical mode duration of 5 s, revealed apical ballooning with perfusion-contraction mismatch. The patient was eventually diagnosed with TTS.

**Figure 4:**
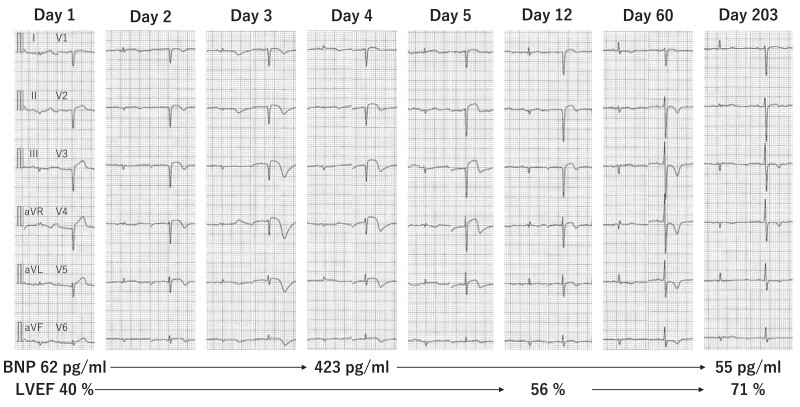
Twelve-lead electrocardiogram, brain natriuretic peptide level, and left ventricular ejection fraction during the follow-up period. BNP, brain natriuretic peptide; LVEF, left ventricular ejection fraction.

After admission to the intensive care unit at 6:45 p.m. on Day 1, the patient was haemodynamically stable under monitoring with a pulmonary artery catheter. Norepinephrine was tapered off. At 11:25 a.m. on Day 3, the Impella device was removed. The 12-lead electrocardiogram showed negative T waves in the leads where ST elevations had been observed. She was discharged on Day 22. On Day 28, cardiac magnetic resonance imaging showed myocardial oedema on T2-weighted imaging but no late gadolinium enhancement in the LV apex. On Day 206, the negative T waves on 12-lead electrocardiography and wall motion abnormalities on echocardiography were significantly lessened (*Figure 4*).

## Discussion

In this case report, biplane LVG during surgical mode was useful for diagnosing TTS by revealing apical ballooning in a patient with ST elevations and worsening cardiogenic shock treated with Impella. Early introduction of mechanical circulatory support (e.g. Impella) in patients with cardiogenic shock has been reported to be associated with improved outcomes.^4,9,10^ If a patient with ST elevations has SCAI stage D or E shock, it is reasonable to introduce Impella before coronary angiography to prioritize treating tissue hypoperfusion.^11^ If there are no lesions in the epicardial coronary arteries that can be explained as a cause of acute coronary syndrome, biplane LVG in similar views can be considered to evaluate for TTS. However, LV unloading with Impella might prevent the contrast media from filling the LV. This report demonstrated that in a patient with ST elevations and refractory cardiogenic shock, biplane LVG with an Impella device in temporary surgical mode could adequately assess LV wall motion abnormalities without affecting haemodynamics. The surgical mode is able to protect the motor because the purge system remains active even when the pump stops. The surgical mode is generally activated to allow aortic cross-clamping and open heart surgery while the device is in the left ventricle. In this case, the device was in surgical mode for only 5 s. If the Impella device is re-driven as soon as LVG is completed, haemodynamics will not be affected. In this patient, LV wall motion abnormalities were accurately determined with LVG, leading to the diagnosis of TTS.

## Conclusion

In patients with TTS complicated by cardiogenic shock who are treated with Impella, LVG during surgical mode allows for accurate assessment of LV wall motion abnormalities.

## Lead author biography


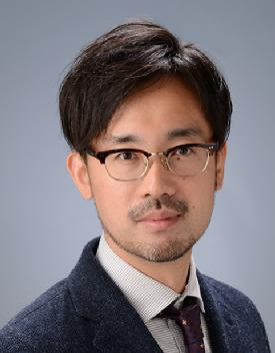


Shoji Kawakami is a double board–certificated interventional cardiologist and intensivist who works for Aso Iizuka Hospital in Japan. He received his medical degree at Oita University in 2006 and obtained a doctor of philosophy in medicine from Kumamoto University in 2017. He worked as an attending doctor in 2014–2018 in the National Cerebral and Cardiovascular Center in Japan.

## Supplementary Material

ytae578_Supplementary_Data

## Data Availability

The data underlying this article will be shared upon reasonable request to the corresponding author.

## Supplementary material

**Consent:** The authors confirm that written consent for submission and publication of this case report has been obtained from the patient in line with the Committee on Publication Ethics guidance.

**Funding:** None declared.
